# LifeWatch observatory data: phytoplankton observations in the Belgian Part of the North Sea

**DOI:** 10.3897/BDJ.8.e57236

**Published:** 2020-12-16

**Authors:** Luz Amadei Martínez, Jonas Mortelmans, Nick Dillen, Elisabeth Debusschere, Klaas Deneudt

**Affiliations:** 1 Flanders Marine Institute (VLIZ), Wandelaarkaai 7, Oostende, Belgium Flanders Marine Institute (VLIZ), Wandelaarkaai 7 Oostende Belgium; 2 Ghent University, Department of Biology, Laboratory of Protistology & Aquatic Ecology, Ghent, Belgium Ghent University, Department of Biology, Laboratory of Protistology & Aquatic Ecology Ghent Belgium

**Keywords:** phytoplankton, Belgium, marine, LifeWatch Belgium, FlowCAM, image recognition

## Abstract

**Background:**

This paper describes a phytoplankton data series generated through systematic observations in the Belgian Part of the North Sea (BPNS). Phytoplankton samples were collected during multidisciplinary sampling campaigns, visiting nine nearshore stations with monthly frequency and an additional eight offshore stations on a seasonal basis.

**New information:**

The data series contain taxon-specific phytoplankton densities determined by analysis with the Flow Cytometer And Microscope (FlowCAM®) and associated image-based classification. The classification is performed by two separate semi-automated classification systems, followed by manual validation by taxonomic experts. To date, 637,819 biological particles have been collected and identified, yielding a large dataset of validated phytoplankton images. The collection and processing of the 2017–2018 dataset are described, along with its data curation, quality control and data storage. In addition, the classification of images using image classification algorithms, based on convolutional neural networks (CNN) from 2019 onwards, is also described. Data are published in a standardised format together with environmental parameters, accompanied by extensive metadata descriptions and finally labelled with digital identifiers for traceability. The data are published under a CC‐BY 4.0 licence, allowing the use of the data under the condition of providing the reference to the source.

## Introduction

Phytoplankton contributes to almost half of the Earth’s total primary production (Field et al. 1998), it is the base of the marine food web and alterations to its composition and abundance often have repercussions on higher trophic levels, including those of economic importance (Richardson and Schoeman 2004). In addition, harmful algal blooms cause economic losses to aquaculture, fisheries and tourism (Hallegraeff 1993, Wells et al. 2020, Anderson et al. 2012). Furthermore, phytoplankton has an important role as carbon pump sequestering carbon dioxide from the surface sinking it in the deep sea (Buesseler et al. 2007, Hutchins and Fu 2017). Due to their small size, short generation times and large population numbers, phytoplankton are indicators of marine ecosystem change (Margalef 1978).

The availability of long-term phytoplankton observational data for the Belgian Part of the North Sea (BPNS) is limited. In the last decades, several studies have described the Belgian phytoplankton community structure (Muylaert et al. 2006, Muylaert et al. 2009, Gasparini et al. 2000, Breton et al. 2006). The 4DEMON project integrated dispersedly-gathered phytoplankton abundance data from research projects in the BPNS between 1968 and 2010 (Nohe et al. 2018). However, as most of the sampling was limited in time and orientated towards single sampling locations, information on the spatial dynamics of the phytoplankton in the BPNS remains scarce (Muylaert et al. 2006).

In general, long-term time series of phytoplankton are hard to come by (Edwards et al. 2001, Suikkanen et al. 2007, Edwards et al. 2010) because its species composition and abundance are highly variable (Suikkanen et al. 2007) and characterising them using traditional methods is tedious, time-consuming and expensive (Lund et al. 1958, Zingone et al. 2015). Over the past decades, there has been a proliferation of imaging systems to count and measure plankton in a faster and more efficient manner (e.g. Cytobuoy, FlowCytobot or FlowCAM) (Benfield et al. 2007, Haraguchi et al. 2018, Álvarez et al. 2014). Digital flow cytometry using FlowCAM® (Fluid Imaging Technologies, Scarborough, Maine U.S.A.) has gained attention as a means of rapid cell counting of phytoplankton since first used by Sieracki et al. (1998).

## General description

### Purpose

In response to the identified data gap for the BPNS and taking into account the availability of the newest imaging technology, a long-term phytoplankton observation effort was initiated as part of the Flemish contribution to LifeWatch. Multidisciplinary sampling campaigns are organised in the BPNS on a regular basis, collecting phytoplankton samples that are processed with a digital imaging flow cytometer (FlowCAM). The procedures put in place for automated processing and manual validation manifest a durable approach for the generation of a long-term high-quality phytoplankton time series.

## Project description

### Title

LifeWatch observatory data: phytoplankton observations by imaging flow cytometry (FlowCam) in the Belgian Part of the North Sea

### Personnel

Deneudt K.; Mortelmans J.; Muyle J.; Debusschere E.; Dillen N.; Amadei Martínez L.

### Study area description

The BPNS is located in the Southern Bight of the North Sea. It is characterised by shallow waters (< 40 m) and strong semi-diurnal tidal currents resulting in a vertically homogeneous water column (Lee 1980, Muylaert et al. 2006). Its waters are influenced by freshwater discharges (from Yzer, Scheldt, Meus, Seine) and saltwater inflow (Atlantic water, coming in through the English Channel), resulting in an on-offshore gradient (Lancelot et al. 1987, Lacroix et al. 2004). In addition, the BPNS is an area heavily impacted by the introduction of non-indigenous species, industrial and agricultural pollution, overfishing and trawling, dredging, human-induced eutrophication, sand and gravel extraction, offshore construction and heavy shipping traffic (Emeis et al. 2015).

### Design description

Stations are visited in the course of one to three-day sampling cruises with the RV Simon Stevin on a monthly or seasonal frequency. Sampling activities onboard are registered in the Marine Information Data Acquisition System (MIDAS). Through MIDAS, scientists can record the metadata of their scientific actions (e.g. time, coordinates, action type, start and stop of the action, station, status of deployment and notes). MIDAS also registers the navigation (heading, current time, latitude, longitude, speed, course over ground, navigation depth and draught), together with meteorological (air temperature, relative humidity, wind direction and speed) and oceanographic data (sea surface water temperature, salinity, chlorophyll-a and sound velocity). This information is synchronised with the VLIZ ICT network every 24 hours and is made available online through the VLIZ website.

## Sampling methods

### Study extent

**Figure 1.**
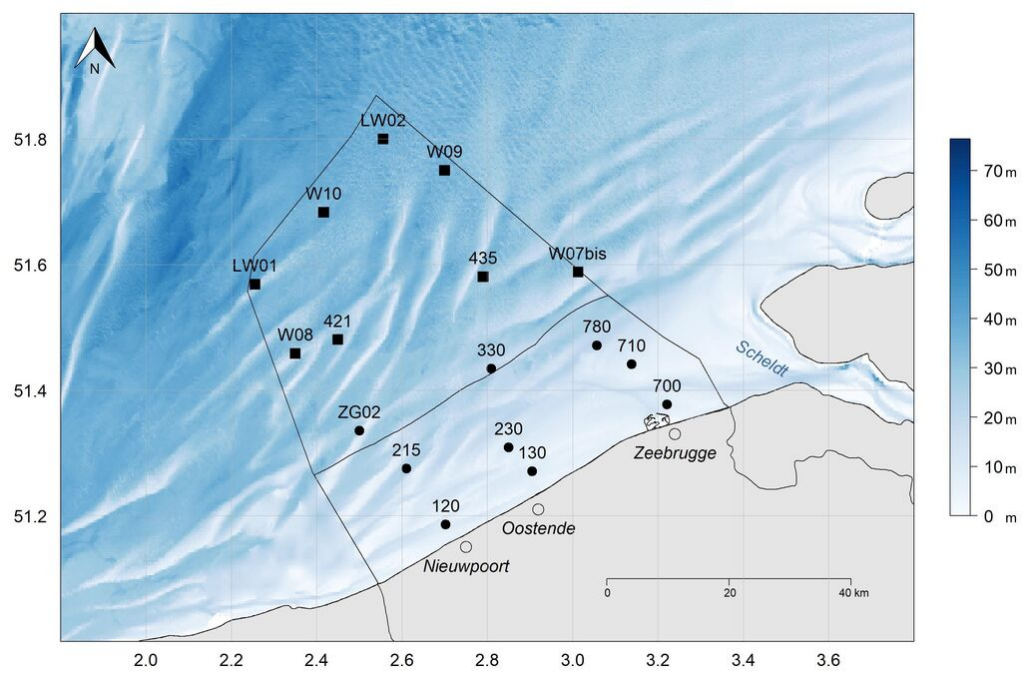
Study sites on the Belgian Part of the North Sea (BPNS). Nine stations onshore (black points), visited monthly: 120 (51°11'9.6", 2°42'9"); 130 (51°16'13.8", 2°54'19.2"); 215 (51°16'29.4", 2°36'39"); 230 (51°18'31.2", 2°51'1.2"); 330 (51°26'3", 2°48'32.4"); ZG02 (51°20'6.6", 2°30'2.4"); 700 (51°22'37.2", 3°13'15.6"); 710 (51°26'28.2", 3°8'18"); 780 (51°28'16.8", 3°3'26.4"); and eight additional offshore stations (black squares), visited seasonally: LW01 (51°34'7.2", 2°15'21.6"); LW02 (51°48'0", 2°33'21.6"); W07bis (51°35'16.8", 3°0'45"); W08 (51°27'30", 2°21'0"); W09 (51°45'0", 2°42'0"); W10 (51°41'0", 2°25'0"); 421 (51°28'49.8", 2°27'0"); and 435 (51°34'50.4", 2°47'25.2"). The 12 nautical mile zone is indicated. The x-axis represents the longitude and the y-axis the latitude in decimal degrees.

**Figure 2.**
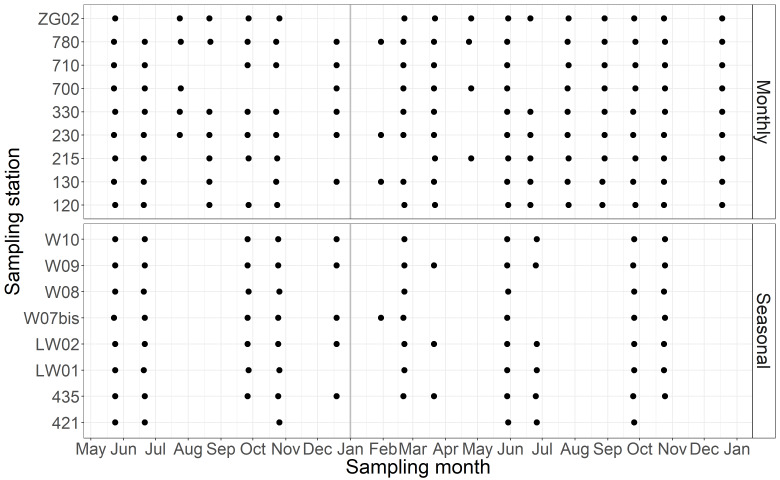
Data availability in the sampled area in the Belgian Part of the North Sea and station name (as described in Design description: monthly campaigns and seasonal campaigns) from May 2017 to December 2018.

A spatial grid of 17 stations, spread over the BPNS, is being sampled since May 2017 (Fig. 1). Nine nearshore stations are sampled on a monthly basis. Eight additional stations, positioned further offshore, are sampled only with seasonal frequency (Fig. 2). The stations are part of the LifeWatch marine observatory (http://www.lifewatch.be) that forms a dense net of sensor networks and observation stations in the Belgian coastal waters and sandbank system, a designated site in the Long Term Ecological Research (LTER) network (Muelbert et al. 2019).

### Sampling description

Surface water samples are collected in every station, fixed with acid Lugol (5%) and stored in cold (4°C) and dark conditions. Once in the lab, samples are processed with the FlowCAM using the 300-µm deep flow cell with the 4X objective, capturing the particles with an Equivalent Spherical Diameter (ESD) between 70 and 300 µm in 2017 and 55-300 µm from 2018 onwards. In 2017 and 2018, using the autoclassification tool of VisualSpreadsheet, the images collected were assigned to a taxon and further, a taxonomist validated the automatic classification. From 2019 onwards, the classification of images is performed using image classification algorithms, based on convolutional neural networks (CNN), using as training set the validated images from 2017 and 2018.

### Quality control

The output of both classification processes are manually validated by an experienced taxonomist to remove the errors of the automatic prediction. In this step, the taxonomist checks that all the imaged particles have been assigned to the correct category by the automatic classification, if not, the particles are manually changed to the right category. The taxonomist evaluates 2 times all the particles to correct the possible misclassifications. The species identification is done with the help of Tomas (1997), Kraberg et al. (2010) and Alfred Wegener Institute for Polar and Marine Research (AWI) (2020). A summary with the morphological description of the categories found in the dataset and example FlowCAM images is available upon request. All manual input towards the databases is guided by forms and fields with associated input rules avoiding the most common editing errors. Taxon names are linked to the corresponding AphiaID’s of WoRMS (WoRMS Editorial Board 2020), hereby linking to the most recent accepted names and authorities.

### Step description

**Sampling at sea**

The phytoplankton samples are collected with a stainless steel bucket. In total, either 50 or 70 litres of surface water are hauled up onboard and poured into an Apstein net (1.2 m long, 55 µm mesh size and 50 cm diameter). The volume of water collected is documented in MIDAS. The sample is concentrated in a plastic jar at the cod-end of the net, where the sample and rinsing water escapes through a 55 µm mesh window. Immediately afterwards, the sample is preserved in acid Lugol’s solution at a 5% final concentration and stored onboard in dark conditions at 4°C. At the end of the sampling campaign, the samples are transported and stored in the Marine Station Ostende (MSO) at 4°C until processing. The remaining sample material after processing is available to researchers for re-use.

**FlowCAM processing**

Within three months after collection, the samples are processed using the FlowCAM VS-4 (Fluid Imaging Technologies, Yarmouth, Maine, U.S.A.) and the software VisualSpreadsheet® Version 4.2.52. FlowCAM combines the technologies of flow cytometry, microscopy and image analysis (Sieracki et al. 1998). It counts and photographs particles moving in a fluid flow. The sample passes through a flow cell, drawn by the associated syringe pump of the particular flow cell. A digital grey-scale camera captures the particles as they pass in front of the microscope (Álvarez et al. 2011). The output is a collection of pictures, combined in collages that constitute the output of VisualSpreadSheet (Álvarez et al. 2012). In addition, a List File contains the particle properties of each targeted particle (Camoying and Yñiguez 2016).

For this dataset, the 300-µm deep flow cell with the 4X objective and the 5 ml syringe pump are used. This combination maximises the taxonomic resolution for the size range of interest without compromising the running time. Sample preservation with Lugol negates the ability to discriminate cells from detritus through the detection of chlorophyll (Graham et al. 2018). Therefore, samples are processed using the AutoImage working mode imaging particles in a user-defined number of frames per second (FPS) (here, 20 FPS) and a flow rate of 1.7 ml min^-1^. The setting of choice in VisualSpreadsheet is a Basic Size Acquisition Filter selecting particles, based on the ESD (70-300 ESD in 2017; and 55-300 ESD from 2018 onwards). The setting of the focus is done directly on the sample, instead of using the focus beads, since this practice is more time effective. Then, a 1.5 ml subsample is run to obtain information on the particle concentration. If the concentration is too high, the sample is diluted to a concentration of < 600 particles ml^-1^ to reduce the chance of overlapping particles in the captured frames.

Attachment of diatoms with spines to the flow cell wall (e.g. *Chaetoceros* Ehrenberg) and aggregation of chain-forming diatoms (e.g. *Bellerochea*) often interfere with the sample processing. To minimise clogging and to increase the durability of the flow cell, each sample is pre-filtered in a 300-µm mesh-size net (Álvarez et al. 2011, Álvarez et al. 2012). A periodic pinch of the flow cell tubing by the operator reduces clogging, thus assuring a constant flow of particles (Poulton and Martin 2010). To reduce the variability, each sample has three technical replicates, each of them capturing a maximum of 1,500 particles or covering a total Sample Volume Processed of 5 ml in 2017 and 8 ml from 2018 onwards. When the sample is processed, the flow cell is cleaned with two cycles of 5 ml of Milli-Q® water; ethanol (70%), leaving little air in between fluids; and finishing with Milli-Q® water.

To convert from cell counts in the FlowCAM to phytoplankton Abundance (cell l^-1^), we used the following formula:

\begin{varwidth}{50in}
        \begin{equation*}
            Abundance (cells/L) = {count \over Vol. imaged (mL) \quad*\quad Dilution factor\quad *\quad Vol. filtered (L) \quad* \quad Vol.sample (mL)}
        \end{equation*}
    \end{varwidth}

were *Abundance* is defined as the number of cells in a litre of the unfiltered water sample, *Vol. imaged* is the volume in the field of view of each sample, *Vol. filtered* is the volume poured into the Apstein net and *Vol. sample* is the remaining sample after the filtration in the Apstein net.

**Semi-automatic classification with VisualSpreadsheet (2017-2018)**

A reference library with phytoplankton images for the Southern Bight of the North Sea is created using the autoclassification tool of VisualSpreadsheet and the manual validation. Following software recommendations, the reference library consists of various categories, each containing 10 - 20 images (regions of interest; ROIs) for each category and covers a species or higher taxon group in case identification at species level is not possible. This is called "class" in the VisualSpreadsheet and, based on those images per library, filters are defined. A category can contain several filters to represent different orientations or developmental stages of the same taxon (e.g. *Chaetoceros* in valve view or girdle view). The combination of categories with its filters are stored as a learning set that is used to run an Auto Classification and assign the sample particles to different categories and taxon groups. In addition, separate library categories are also created for non-phytoplanktonic particles (e.g. crustacea, eggs, detritus…). Due to the large diversity of taxa in the samples and the variation in species composition over the year, the combination of used categories in the learning set needs to be adapted regularly. Only the categories of the taxa expected to be present are used. Categories with its filters are applied following the order of the most abundant taxa to least abundant. The obtained classification is validated manually by taxonomic experts.

**Semi-automatic classification with CNNs (2019 - current)**

Since 2019, the classification of our FlowCAM images is facilitated by using deep learning classifiers, more specifically CNNs. One of the prerequisites for allowing the use of deep learning classifiers is the availability of a large training dataset. Once our validated FlowCAM dataset (2017-2018) was sufficiently large, it became possible to shift towards CNNs for class prediction of the images. The main benefit of using CNNs is the increased classification accuracy, reducing the time spent by trained taxonomists to validate the data afterwards. Consequently, this also allows the data to be released to the public sooner.

The current iteration of the CNN in use is the one provided and trained by Instituto de Física de Cantabria (IFCA, Spain) (Lloret et al. 2018). The classifier is trained in detecting 53 microplankton classes, compromising 42 genera. The training dataset was sampled from the entire FlowCAM dataset, but limiting the maximum number of images per category at 30,000. For every category, 90% of the images were used as training data and 5% each for validating and testing. The trained model predicts for each image the probability it belongs to each defined category. By using the prediction with the highest probability, the current CNN approach reaches a classification accuracy of 90.7%. A 99.4% accuracy is reached when allowing the correct label to be in the top five highest probability predictions. However, there are still difficulties with the classification of rare taxa that hold hardly any validated ROIs. These rare taxa prevent the use of this classifier as a fully autonomous classification system. Human validation remains therefore imperative.

Moving towards a new classification methodology also offers opportunities to further automate and standardise our FlowCAM data processing pipeline. In the new setup, raw output files from the FlowCAM are directly processed by a set of python scripts. The typical “FlowCAM-collages” are cropped into separate ROIs, a clean data table describing all ROIs is generated and additional sample processing metadata is incorporated into the output directory. This avoids the use of VisualSpreadsheet, allowing more and easy control over the data, as well as enabling automation of the dataflow. The generated files are uploaded to a MongoDB server where they are classified by the CNN.

## Geographic coverage

### Description

Data were collected in 17 stations over the BPNS (Fig. 1).

### Coordinates

51°5'21.5"N and 51°52'34"N Latitude; 3°22'13.4"E and 2°14'8"E Longitude.

## Taxonomic coverage

### Description

The dataset is composed of 55 categories identified at species level or higher taxon group if the identification at species level is not possible. Bacillariophyceae (33 taxa) and Dinophyceae (7 taxa) are the most abundant phytoplankton classes in the dataset, the rest of the dataset being formed by non-phytoplanktonic categories (15).

**Figure 3.**
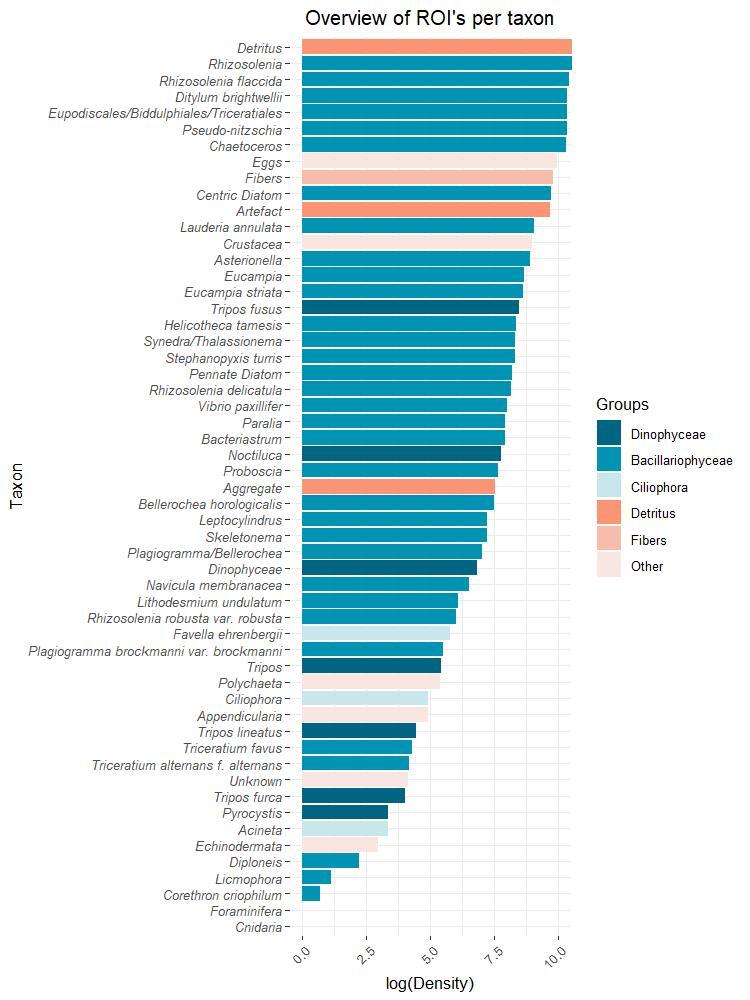
Cumulative log transformed density (cell l^-1^) per taxon in the sampled area in the Belgian Part of the North Sea.

The validated dataset shows that, from May 2017 to December 2018, diatoms (Bacillariophyceae) (310,132 ROIs) such as *Rhizosolenia* (117183 ROIs), *Guinardia flaccida* (32,486 ROIs), *Pseudo-nitzschia* (28,285 ROIs) and *Ditylum brightwellii* (24,989 ROIs) are the most abundant taxa in the sampling period. In the case of dinoflagellates (Dinophyceae) (6,044 ROIs), *Tripos fusus* (4,616 ROIs) is the most abundant species (Fig. 3).

### Taxa included

**Table:** 

Rank	Scientific Name	
class	* Appendicularia *	
species	*Corethron criophilum* Castracane, 1886	
genus	*Licmophora* C.A. Agardh, 1827	
genus	*Diploneis* (C. G. Ehrenberg) P.T. Cleve, 1894	
species	*Plagiogramma vanheurckii* Grunow, 1881	
species	Triceratium alternans f. alternans J.W. Bailey, 1851	
genus	*Leptocylindrus* P.T. Cleve in C.G.J. Petersen, 1889	
species	*Triceratium favus* Ehrenberg, 1839	
genus	Plagiogramma / Bellerochea	
species	Plagiogramma brockmanni var. brockmanni Hustedt, 1939	
species	*Lithodesmium undulatum* Ehrenberg, 1839	
species	Rhizosolenia robusta var. robusta Norman ex Ralfs in Pritchard, 1861	
species	*Navicula membranacea Cleve, 1897*	
genus	*Skeletonema* R.K. Greville, 1865	
genus	*Proboscia* B.G. Sundstrom, 1986	
genus	*Asterionella* A.H. Hassall, 1850	
genus	*Bacteriastrum* G. Shadbolt, 1854	
species	*Rhizosolenia delicatula* Cleve, 1900	
genus	*Paralia* P.A.C. Heiberg, 1863	
species	*Bellerochea horologicalis* Stosch, 1980	
species	*Vibrio paxillifer* O.F.Müller, 1786	
species	*Stephanopyxis turris* (Greville) Ralfs, 1861	
species	*Helicotheca tamesis* (Shrubsole) M.Ricard, 1987	
genus	*Synedra / Thalassionema*	
genus	*Eucampia* C.G. Ehrenberg, 1839	
species	*Eucampia striata* Stolterfoth, 1879	
species	*Lauderia annulata* Cleve, 1873	
genus	*Chaetoceros* C.G. Ehrenberg, 1844	
order	*Eupodiscales / Biddulphiales / Triceratiales*	
species	*Ditylum brightwellii* (T.West) Grunow, 1885	
genus	*Pseudo-nitzschia* H. Peragallo in H. Peragallo & M. Peragallo, 1900	
species	*Rhizosolenia flaccida* Castracane, 1886	
genus	*Rhizosolenia* T. Brightwell, 1858	
genus	*Acineta* Ehrenberg, 1834	
species	*Favella ehrenbergii* (Claparède & Lachmann, 1858) Jörgensen, 1924	
subphylum	Crustacea	
genus	*Pyrocystis* J.Murray ex Haeckel, 1890	
species	*Tripos fusus* (Ehrenberg) F.Gómez, 2013	
species	*Tripos lineatus* (Ehrenberg) F.Gómez, 2013	
genus	*Tripos* Bory de Saint-Vincent, 1823	
class	Dinophyceae	
genus	*Noctiluca* Suriray, 1836	
phylum	Foraminifera	
phylum	Cnidaria	
phylum	Echinodermata	
class	Polychaeta	

## Temporal coverage

**Data range:** 2017-5-08 – 2018-12-18.

### Notes

See Fig. 2

## Usage licence

### Usage licence

Open Data Commons Attribution License

### IP rights notes

The dataset is licensed under a Creative Commons CC-BY4.0 licence, allowing the use of the data under the condition of providing the reference to the original source. When using the data in publications, acknowledgement of LifeWatch is required. This can be done by adding the reference to the used dataset version; for example, the used “Flanders Marine Institute (VLIZ), Belgium (2020): LifeWatch observatory data: phytoplankton observations by imaging flow cytometry (FlowCAM) in the Belgian Part of the North Sea. https://doi.org/10.14284/424 and by referring to the current data paper.

## Data resources

### Data package title

LifeWatch observatory data: phytoplankton observations by imaging flow cytometry (FlowCam) in the Belgian Part of the North Sea

### Resource link

https://doi.org/10.14284/424

### Number of data sets

3

### Data set 1.

#### Data set name

event.txt

#### Data format

Tab delimited Darwin Core Archive

#### Number of columns

20

#### Character set

UTF-8

#### 

**Data set 1.** 

Column label	Column description
id	An identifier for the set of information associated with an Event (something that occurs at a place and time). May be a global unique identifier or an identifier specific to the dataset.
type	The nature or genre of the resource.
modified	The most recent date-time on which the resource was changed.
language	The language of the resource.
rightsHolder	A person or organisation owning or managing rights over the resource.
accessRights	Information about who can access the resource or an indication of its security status. Access Rights may include information regarding access or restrictions based on privacy, security, or other policies.
datasetName	The name identifying the dataset from which the record was derived.
ownerInstitutionCode	The name (or acronym) in use by the institution having ownership of the object(s) or information referred to in the record.
eventID	An identifier for the set of information associated with an Event (something that occurs at a place and time). May be a global unique identifier or an identifier specific to the dataset.
parentEventID	An identifier for the broader Event that groups this and potentially other Events.
samplingProtocol	The method or protocol used during an Event.
eventDate	The date-time or interval during which an Event occurred. For occurrences, this is the date-time when the event was recorded. Not suitable for a time in a geological context.
locationID	An identifier for the set of location information (data associated with dcterms:Location). May be a global unique identifier or an identifier specific to the dataset.
waterBody	The name of the water body in which the Location occurs.
country	The name of the country or major administrative unit in which the Location occurs.
countryCode	The standard code for the country in which the Location occurs.
minimumDepthInMeters	The lesser depth of a range of depth below the local surface, in metres.
maximumDepthInMeters	The greater depth of a range of depth below the local surface, in metres.
decimalLatitude	The geographic latitude (in decimal degrees, using the spatial reference system given in geodeticDatum) of the geographic centre of a Location. Positive values are north of the Equator, negative values are south of it. Legal values lie between -90 and 90, inclusive.
decimalLongitude	The geographic longitude (in decimal degrees, using the spatial reference system given in geodeticDatum) of the geographic centre of a Location. Positive values are east of the Greenwich Meridian, negative values are west of it. Legal values lie between -180 and 180, inclusive.

### Data set 2.

#### Data set name

EMOF

#### Number of columns

11

#### Character set

UTF-8

#### 

**Data set 2.** 

Column label	Column description
id	An identifier for the MeasurementOrFact (information pertaining to measurements, facts, characteristics or assertions). May be a global unique identifier or an identifier specific to the dataset.
occurrenceID	An identifier for the Occurrence (as opposed to a particular digital record of the occurrence). In the absence of a persistent global unique identifier, construct one from a combination of identifiers in the record that will most closely make the occurrenceID globally unique.
measurementType	The nature of the measurement, fact, characteristic or assertion.
measurementTypeID	An identifier for the nature of the measurement, fact, characteristic or assertion.
measurementValue	The value of the measurement, fact, characteristic or assertion.
measurementValueID	An identifier for the value of the measurement, fact, characteristic or assertion.
measurementUnit	The units associated with the measurementValue.
measurementUnitID	An identifier for the units associated with the measurementValue.
measurementDeterminedBy	A list (concatenated and separated) of names of people, groups or organisations who determined the value of the MeasurementOrFact.
measurementMethod	A description of or reference to (publication, URI) the method or protocol used to determine the measurement, fact, characteristic or assertion.
measurementRemarks	Comments or notes accompanying the MeasurementOrFact.

### Data set 3.

#### Data set name

Occurence

#### Number of columns

8

#### Character set

UTF-8

#### 

**Data set 3.** 

Column label	Column description
id	An identifier for the Occurrence (as opposed to a particular digital record of the occurrence). In the absence of a persistent global unique identifier, construct one from a combination of identifiers in the record that will most closely make the occurrenceID globally unique.
modified	The most recent date-time on which the resource was changed.
basisOfRecord	The specific nature of the data record.
occurrenceID	An identifier for the Occurrence (as opposed to a particular digital record of the occurrence). In the absence of a persistent global unique identifier, construct one from a combination of identifiers in the record that will most closely make the occurrenceID globally unique.
occurrenceStatus	A statement about the presence or absence of a Taxon at a Location.
eventID	An identifier for the set of information associated with an Event (something that occurs at a place and time). May be a global unique identifier or an identifier specific to the dataset.
scientificNameID	An identifier for the nomenclatural (not taxonomic) details of a scientific name.
scientificName	The full scientific name, with authorship and date information, if known. When forming part of an Identification, this should be the name in lowest level taxonomic rank that can be determined. This term should not contain identification qualifications, which should instead be supplied in the IdentificationQualifier term.

## Additional information

### Dataset location and format

**Figure 4.**
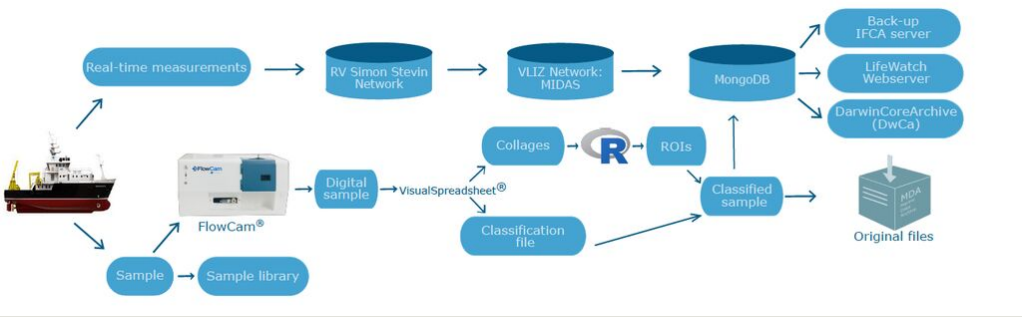
Schematic overview of the data-flow from ship to user, before 2019.

**Figure 5.**
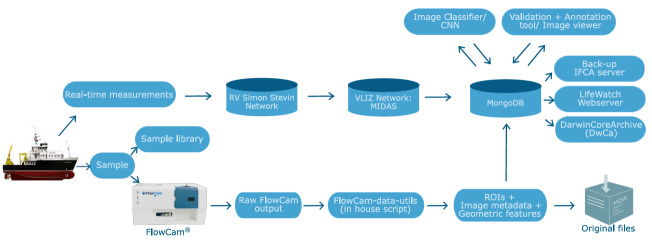
Schematic overview of the data-flow from ship to user, since 2019.

Data are made available through the LifeWatch data explorer (Flanders Marine Institute 2020) where users can access, visualise and download the quality-controlled data table that includes the Trip action ID, Date (Time), Station, Taxon, Abundance (Density) and additional metadata. Each sample, with its unique Trip action ID, presents several rows, one for the abundance of each Taxon. In the background, all particle data including cropped pictures, taxonomic annotation and associated sample and particle metadata are stored in a MongoDB data system and are not downloadable, but they are accessible upon request. This database is replicated as a back-up on servers of Instituto de Física de Cantabria (IFCA) in Santander, Spain. For long-term preservation, the original data files are archived in the Marine Data Archive. The quality-controlled classification files and the cropped pictures of the FlowCAM are archived to a network archive on the VLIZ servers and linked to its metadata in the MIDAS system. This database is uploaded on to the IFCA server (Santander, Spain). For further redistribution and exchange with European and global data systems, the data are integrated in the European node of the Ocean Biogeographic Information System (EurOBIS) and the Biology portal of the European Marine Observation and Data Network (EMODnet). The inputs to these networks are currently done through yearly exports, but procedures enabling higher data exchange frequencies are under development. The data exchange requires reformatting in accordance with the OBIS-ENV DATA format, which is an adaptation of the Darwin Core Archive (DwC-A) schema, developed for sample-based marine biological data (De Pooter et al. 2017). In the OBIS-ENV DATA standard, the DwC-A file contains three main structural elements: an Event core linked to an Occurrence extension and an ExtendedMeasurementOrFact extension (eMoF). The Event core stores information on sampling location, time and depth. The Occurrence extension stores the presence/absence data of the taxa. The EMoF contains the abundance data, the environmental data at the moment of the sampling, the sampling equipment and the protocols. The EMOF data is standardised following controlled vocabularies managed by the British Oceanographic Datacentre and the European SeaDataNet project. Fixed versions of the database are distributed annually (e.g. Flanders Marine Institute 2020). A metadata record is created in the dataset catalogue of the Integrated Marine Information System and dataset versions are labelled with a Digital Object Identifier (DOI). The complete data pathway is given in Fig. 4 (pre-2019) and Fig. 5 (post-2019).

### Current usage and future perspectives

Monitoring of phytoplankton via the FlowCAM is part of a long term ESFRI initiative. Regular updates of the validated data are accessible on the LifeWatch data explorer and a yearly dataset is published on MDA. Valorisation of this data is ongoing in the framework of MSFD and in light of the blue economy supporting research, for example, fouling management, nature-based solutions, aquaculture etc. and is part of an artificial intelligence application study.

## References

[B6151325] (AWI) Alfred Wegener Institute for Polar and Marine Research http://planktonnet.awi.de.

[B5814127] Álvarez E., López-Urrutia Á., Nogueira E., Fraga S. (2011). How to effectively sample the plankton size spectrum? A case study using FlowCAM.. Journal of Plankton Research.

[B5814155] Álvarez E., López-Urrutia Á., Nogueira E. (2012). Improvement of plankton biovolume estimates derived from image-based automatic sampling devices: application to FlowCAM. Journal of Plankton Research.

[B6149855] Álvarez Eva, Moyano Marta, López-Urrutia Ángel, Nogueira Enrique, Scharek Renate (2014). Routine determination of plankton community composition and size structure: a comparison between FlowCAM and light microscopy. Journal of Plankton Research.

[B6149783] Anderson D. M., Cembella A. D., Hallegraeff G. M. (2012). Progress in understanding harmful algal blooms: paradigm shifts and new technologies for research, monitoring, and management. Annual Review of Marine Science.

[B5814167] Benfield M. C., Grosjean P., Culverhouse P. F., Irigoien X., Sieracki M. E., Lopez-Urrutia A., Dam H. G., Hu Q., Davis C. S., Hansen A., Pilskaln C. H., Riseman E. M., Schultz H., Utgoff P. E., Gorsky G. (2007). RAPID: Research on Automated Plankton Identification.. Oceanography.

[B5814208] Breton Elsa, Rousseau Véronique, Parent Jean-Yves, Ozer José, Lancelot Christiane (2006). Hydroclimatic modulation of diatom/*Phaeocystis* blooms in nutrient-enriched Belgian coastal waters (North Sea). Limnology and Oceanography.

[B5814219] Buesseler K. O., Lamborg C. H., Boyd P. W., Lam P. J., Trull T. W., Bidigare R. R., Bishop J. K. B., Casciotti K. L., Dehairs F., Elskens M., Honda M., Karl D. M., Siegel D. A., Silver M. W., Steinberg D. K., Valdes J., Van Mooy B., Wilson S. (2007). Revisiting carbon flux through the ocean's twilight zone. Science.

[B5814243] Camoying Marianne G., Yñiguez Aletta T. (2016). FlowCAM optimization: Attaining good quality images for higher taxonomic classification resolution of natural phytoplankton samples. Limnology and Oceanography: Methods.

[B5840715] De Pooter Daphnis, Appeltans Ward, Bailly Nicolas, Bristol Sky, Deneudt Klaas, Eliezer Menashè, Fujioka Ei, Giorgetti Alessandra, Goldstein Philip, Lewis Mirtha, Lipizer Marina, Mackay Kevin, Marin Maria, Moncoiffé Gwenaëlle, Nikolopoulou Stamatina, Provoost Pieter, Rauch Shannon, Roubicek Andres, Torres Carlos, van de Putte Anton, Vandepitte Leen, Vanhoorne Bart, Vinci Matteo, Wambiji Nina, Watts David, Klein Salas Eduardo, Hernandez Francisco (2017). Toward a new data standard for combined marine biological and environmental datasets - expanding OBIS beyond species occurrences.. Biodiversity Data Journal.

[B5814253] Edwards M., Reid P., Planque P. (2001). Long-term and regional variability of phytoplankton biomass in the Northeast Atlantic (1960–1995). ICES Journal of Marine Science.

[B6149801] Edwards M., Beaugrand G., Hays G. C., Koslow J. A., Richardson A. J. (2010). Multi-decadal oceanic ecological datasets and their application in marine policy and management. Trends in Ecology & Evolution.

[B5814263] Emeis Kay-Christian, van Beusekom Justus, Callies Ulrich, Ebinghaus Ralf, Kannen Andreas, Kraus Gerd, Kröncke Ingrid, Lenhart Hermann, Lorkowski Ina, Matthias Volker, Möllmann Christian, Pätsch Johannes, Scharfe Mirco, Thomas Helmuth, Weisse Ralf, Zorita Eduardo (2015). The North Sea — A shelf sea in the Anthropocene. Journal of Marine Systems.

[B5814285] Field C. B., Behrenfeld M., Randerson J. T., Falkowski P. (1998). Primary production of the biosphere: Integrating terrestrial and oceanic components. Science.

[B6150743] Institute Flanders Marine http://www.lifewatch.be/en/lifewatch-data-explorer.

[B6150594] Institute Flanders Marine (2020). LifeWatch observatory data: phytoplankton observations by imaging flow cytometry (FlowCam) in the Belgian Part of the North Sea..

[B5814330] Gasparini S, Daro M. H, Antajan E, Tackx M, Rousseau V, Parent J - Y, Lancelot C (2000). Mesozooplankton grazing during the *Phaeocystis
globosa* bloom in the southern bight of the North Sea. Journal of Sea Research.

[B5814343] Graham M. D., Cook J., Graydon J., Kinniburgh D., Nelson H., Pilieci S., Vinebrooke R. D. (2018). High-resolution imaging particle analysis of freshwater cyanobacterial blooms. Limnology and Oceanography: Methods.

[B5814356] Hallegraeff G. M. (1993). A review of harmful algal blooms and their apparent global increase. Phycologia.

[B6149846] Haraguchi Lumi, Jakobsen Hans H., Lundholm Nina, Carstensen Jacob (2018). Phytoplankton community dynamic: A driver for ciliate trophic strategies. Frontiers in Marine Science.

[B6149792] Hutchins D. A., Fu F. (2017). Microorganisms and ocean global change. Nature Microbiology.

[B6151303] Kraberg A., Baumann M., Dürselen C. (2010). Coastal phytoplankton: photo guide for Northern European seas..

[B5814366] Lacroix Geneviève, Ruddick Kevin, Ozer José, Lancelot Christiane (2004). Modelling the impact of the Scheldt and Rhine/Meuse plumes on the salinity distribution in Belgian waters (southern North Sea). Journal of Sea Research.

[B5814514] Lancelot C., Gilles B., Alain S., Weisse T., Colijn F. (1987). Phaeocystis blooms and nutrient enrichment in the continental coastal zones of the North Sea. Deep Sea Research Part B. Oceanographic Literature Review.

[B5814376] Lee A. J. (1980). North Sea: Physical Oceanography. The North-West European shelf seas: The sea bed and the sea in motion II. physical and chemical oceanography, and physical resources.

[B6311997] Lloret Lara, Heredia Ignacio, Aguilar Fernando, Debusschere Elisabeth, Deneudt Klaas, Hernandez Francisco (2018). Convolutional Neural Networks for Phytoplankton identification and classification. Biodiversity Information Science and Standards.

[B5814386] Lund J. W. G., Kipling C., Le Cren E. D. (1958). The inverted microscope method of estimating algal numbers and the statistical basis of estimations by counting. Hydrobiologia.

[B5814504] Margalef R. (1978). Life‐forms of phytoplankton as survival alternatives in an unstable environment. Oceanologica Acta.

[B6310052] Muelbert J. H., Nidzieko N. J., Acosta A. T.R., Beaulieu S., Bernardino A. F., Boikova E. (2019). ILTER-the international long-term ecological research network as a platform for global coastal and ocean observation. Frontiers in Marine Science.

[B5814396] Muylaert Koenraad, Gonzales Rhia, Franck Melanie, Lionard Marie, Van der Zee Claar, Cattrijsse André, Sabbe Koen, Chou Lei, Vyverman Wim (2006). Spatial variation in phytoplankton dynamics in the Belgian coastal zone of the North Sea studied by microscopy, HPLC-CHEMTAX and underway fluorescence recordings. Journal of Sea Research.

[B5814411] Muylaert Koenraad, Sabbe Koen, Vyverman Wim (2009). Changes in phytoplankton diversity and community composition along the salinity gradient of the Schelde estuary (Belgium/The Netherlands). Estuarine, Coastal and Shelf Science.

[B5814525] Nohe Anja, Knockaert Carolien, Goffin Annelies, Dewitte Elien, De Cauwer Karien, Desmit Xavier, Vyverman Wim, Tyberghein Lennert, Lagring Ruth, Sabbe Koen (2018). Marine phytoplankton community composition data from the Belgian part of the North Sea, 1968–2010. Scientific Data.

[B6149837] Poulton N. J., Martin J. L. (2010). Imaging flow cytometry for quantitative phytoplankton analysis-FlowCAM. Microscopic and molecular methods for quantitative phytoplankton analysis.

[B5813579] Richardson A. J., Schoeman D. S. (2004). Climate impact on plankton ecosystems in the Northeast Atlantic. Science.

[B5814474] Sieracki CK, Sieracki ME, Yentsch CS (1998). An imaging-in-flow system for automated analysis of marine microplankton. Marine Ecology Progress Series.

[B5814484] Suikkanen Sanna, Laamanen Maria, Huttunen Maija (2007). Long-term changes in summer phytoplankton communities of the open northern Baltic Sea. Estuarine, Coastal and Shelf Science.

[B6151278] Tomas C. R. (1997). Identifying marine phytoplankton.

[B6149759] Wells Mark L., Karlson Bengt, Wulff Angela, Kudela Raphael, Trick Charles, Asnaghi Valentina, Berdalet Elisa, Cochlan William, Davidson Keith, De Rijcke Maarten, Dutkiewicz Stephanie, Hallegraeff Gustaaf, Flynn Kevin J., Legrand Catherine, Paerl Hans, Silke Joe, Suikkanen Sanna, Thompson Peter, Trainer Vera L. (2020). Future HAB science: Directions and challenges in a changing climate. Harmful Algae.

[B5838057] WoRMS Editorial Board World Register of Marine Species.. http://www.marinespecies.org.

[B6149824] Zingone Adriana, Harrison Paul J., Kraberg Alexandra, Lehtinen Sirpa, McQuatters-Gollop Abigail, O'Brien Todd, Sun Jun, Jakobsen Hans H. (2015). Increasing the quality, comparability and accessibility of phytoplankton species composition time-series data. Estuarine, Coastal and Shelf Science.

