# Corrigendum to "LifeWatch observatory data: phytoplankton observations in the Belgian Part of the North Sea"

**DOI:** 10.3897/BDJ.10.e81208

**Published:** 2022-02-24

**Authors:** Luz Amadei Martínez, Jonas Mortelmans, Nick Dillen, Elisabeth Debusschere, Klaas Deneudt

**Affiliations:** 1 Flanders Marine Institute (VLIZ), Wandelaarkaai 7, Oostende, Belgium Flanders Marine Institute (VLIZ), Wandelaarkaai 7 Oostende Belgium; 2 Ghent University, Department of Biology, Laboratory of Protistology & Aquatic Ecology, Ghent, Belgium Ghent University, Department of Biology, Laboratory of Protistology & Aquatic Ecology Ghent Belgium

## Note

In the article by Amadei Martínez et al. (2020), the formula used to calculate phytoplankton densities was found erroneous. This formula led to the overestimation of densities in the dataset releases of Flanders Marine Institute (Flanders Marine Institute (VLIZ), Belgium 2019, Flanders Marine Institute (VLIZ), Belgium 2020a, Flanders Marine Institute (VLIZ), Belgium 2020b).

The formula has been updated and the entire dataset has been rectified. Recent dataset releases, i.e. Flanders Marine Institute (Flanders Marine Institute (VLIZ), Belgium 2021) and subsequent, will use the following formula to calculate phytoplankton densities:

\begin{varwidth}{50in}
        \begin{equation*}
            Abundance ({cell\over L}) ={Count*Vol.sample (mL)\over Vol.imaged (mL)*Dilution factor*Vol.filtered (L)}
        \end{equation*}
    \end{varwidth}
